# Pituitary Hyperplasia, Hormonal Changes and Prolactinoma Development in Males Exposed to Estrogens—An Insight From Translational Studies

**DOI:** 10.3390/ijms21062024

**Published:** 2020-03-16

**Authors:** Branka Šošić-Jurjević, Vladimir Ajdžanović, Dragana Miljić, Svetlana Trifunović, Branko Filipović, Sanja Stanković, Sergey Bolevich, Vladimir Jakovljević, Verica Milošević

**Affiliations:** 1 Institute for Biological Research "Siniša Stanković"—National Institute of Republic of Serbia, University of Belgrade, 11060 Belgrade, Serbia; avlada@ibiss.bg.ac.rs (V.A.); lanat@ibiss.bg.ac.rs (S.T.); brankof@ibiss.bg.ac.rs (B.F.); dimi@ibiss.bg.ac.rs (V.M.); 2 Clinic for Endocrinology, Diabetes and Diseases of Metabolism, Clinical Center of Serbia, Faculty of Medicine, University of Belgrade, 11000 Belgrade, Serbia; dragana.miljic@med.bg.ac.rs; 3 Center for Medical Biochemistry, Clinical Centre of Serbia, 11000 Belgrade, Serbia; sanjast2013@gmail.com; 4 Department of Human Pathology, Moscow State Medical University IM Sechenov, 119146 Moscow, Russia; bolevich2011@yandex.ru (S.B.); drvladakgbg@yahoo.com (V.J.); 5 Department of Physiology, Faculty of Medical Sciences, University of Kragujevac, 34000 Kragujevac, Serbia

**Keywords:** pituitary gland, estrogen, prolactinoma, men, rat, microenvironment, folliculo-stellate cells, prolactin, thyroid-stimulating hormone, adrenocorticotropic hormone

## Abstract

Estrogen signaling plays an important role in pituitary development and function. In sensitive rat or mice strains of both sexes, estrogen treatments promote lactotropic cell proliferation and induce the formation of pituitary adenomas (dominantly prolactin or growth-hormone-secreting ones). In male patients receiving estrogen, treatment does not necessarily result in pituitary hyperplasia, hyperprolactinemia or adenoma development. In this review, we comprehensively analyze the mechanisms of estrogen action upon their application in male animal models comparing it with available data in human subjects. Sex-specific molecular targets of estrogen action in lactotropic (PRL) cells are highlighted in the context of their proliferative and secretory activity. In addition, putative effects of estradiol on the cellular/tumor microenvironment and the contribution of postnatal pituitary progenitor/stem cells and transdifferentiation processes to prolactinoma development have been analyzed. Finally, estrogen-induced morphological and hormone-secreting changes in pituitary thyrotropic (TSH) and adrenocorticotropic (ACTH) cells are discussed, as well as the putative role of the thyroid and/or glucocorticoid hormones in prolactinoma development, based on the current scarce literature.

## 1. Introduction

The anterior pituitary is the principal regulator of endocrine homeostasis and is populated by at least five highly differentiated hormone-producing cell types, through which this gland responds to central and peripheral signals. Terminal differentiation and proliferation of lactotropic cells, but also gonadotrophic lineage during embryonic development, is regulated by estrogens [1,2]. In adults, estrogens are able to induce formation of pituitary adenomas in sensitive rat or mice strains, most of which are functional, prolactin (PRL)- and/or growth-hormone (GH)-secreting tumors [3]. In humans, prolonged estrogen treatment does not necessarily result in hyperprolactinemia, pituitary hyperplasia or progression to prolactinomas [4,5]. On the other hand, some case reports indicate the development of prolactinoma in male-to-female transsexuals who are subjected to estrogen treatment [6].

Similar prevalence, but a higher incidence of pituitary prolactinoma formation is observed in women at a younger age in comparison with men [7,8] and disappears after menopause [9]. Moreover, male patients have a higher serum PRL level and larger tumor volume in comparison with female patients with prolactinoma [10,11]. Typically, prolactinomas have a more aggressive clinical behavior in men [12,13]. To date, these sex-related differences have not been fully understood. Most recent basic research, using single-cell genomic approaches, has shed some light on sex-specific expression of genes in lactotropic cells that may, at least in part, underlie the observed differences [14,15,16]. 

The presence of estradiol in males has been known for over 90 years, and it is now well-accepted that estradiol plays an essential role in male physiology [17,18]. Moreover, various estradiol-based pharmacotherapeutics, as well as estrogen-like natural compounds, have found application in men, either in targeted therapy of specific pathological conditions or in alleviation of a whole range of health issues. Androgen deprivation therapy, which is achieved by medical or surgical castration as well as by estrogen therapy, has been widely used in the treatment of prostate cancer for many decades [19]. Moreover, male-to-female transsexual patients use estrogen therapy to adapt their physical bodies to those of the female sex [20,21]. Though estrogen therapy can induce gynecomastia in men, its transdermal or parenteral route of administration was reported to reduce the risk of toxicity [22,23]. Studies examining prolactinoma development in transsexual patients receiving combined antiandrogen and estrogen treatments are confounding, indicating both prolactinoma development [20,24] and/or no influence of estrogen [4]. Clinical data describing the consequences of cross-sexual hormonal treatments on the pituitary, particularly in cases of uncontrolled self-administration or exposure at older age are still lacking [25]. 

In this review, we analyze the key mechanisms through which estrogen treatments may affect the proliferative and secretory activity of pituitary lactotropic cells in male rodent models, by emphasizing sensitive molecular targets of estrogen action expressed in a sex-related manner. Moreover, putative effects of estradiol on the cellular microenvironment and the contribution of postnatal pituitary progenitor/stem cells and transdifferentiation processes to prolactinoma development have also been analyzed. Special attention is given to the less well-studied. pituitary thyrotropic (TSH) and adrenocorticotropic (ACTH) cells in this context and to the putative role of the thyroid and glucocorticoid hormones in prolactinoma development. 

## 2. Estrogen Signaling in the Anterior Pituitary and Prolactinoma Development in Males

Pituitary adenomas are mainly benign tumors with a favorable prognosis that arise from the anterior pituitary. However, they may be clinically relevant, causing a variety of clinical symptoms and associated syndromes due to the overproduction of hormones, and/or have invasive behavior [26], leading to significant morbidity and mortality [27]. 

The molecular mechanisms underlying the generation of pituitary adenomas are largely unknown, but it is clear that estrogen plays an important role in their pathogenesis in both sexes [28]. In males, endogenous estradiol production requires aromatase (CYP19A1), a ubiquitous cytochrome P450 reductase enzyme, which catalyzes local tissue conversion of androgens to estrogens. Aromatase expression has been confirmed in rodent and human pituitaries [29,30,31]. Studies of aromatase overexpression and knockout transgenic mice models confirmed that estradiol is largely responsible for the maintenance of the population of lactotropic cells in males [32,33], apart from playing an essential role in the hypothalamus–pituitary regulation of testicular and associated functions [18,34]. With regard to aromatase expression in pituitary adenomas and its possible association with tumor subtype, sex and/or tumor size, the results are still confounding [29,35,36,37] and await further multimethodological confirmation and testing. Of note, clinical management of hypogonadism in men with macroprolactinoma, which was refractive to dopamine agonists, required aromatase inhibitor treatment in addition to testosterone replacement, to prevent the secondary rise in PRL and, finally, the potential for tumor enlargement [38].

**Figure 1:**
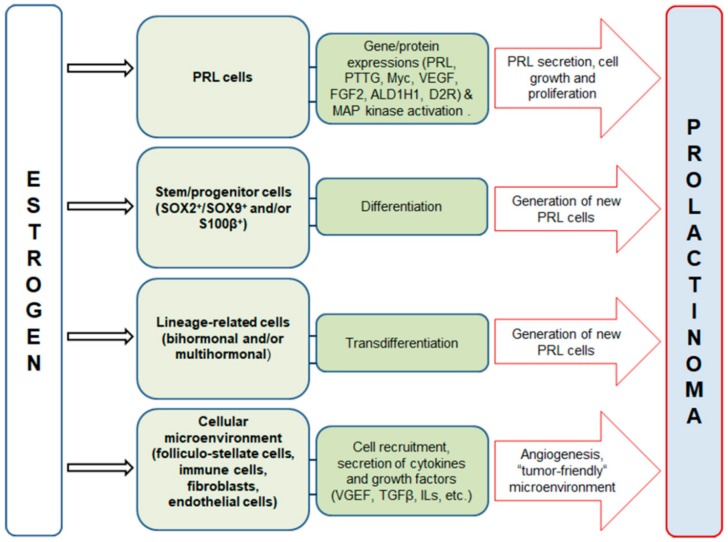
Proposed cellular and molecular targets of estrogen treatment action that contribute to pituitary lactotropic (PRL) cell hyperplasia, hyperprolactinemia and prolactinoma development. Estrogen may affect already-differentiated PRL cells, stem/progenitor cells, lineage-related cells and cells comprising the microenvironment. Transcriptional regulation of the following genes is mainly associated with tumorigenic action of estrogen in the pituitary: pituitary tumor transforming gene (PTTG); Myc; aldehyde dehydrogenase 1A1 (ALDH1A1); D2R, dopamine D2 receptor; mitogen-activated protein kinases (MAPK); vascular endothelial growth factor (VEGF); fibroblast growth factor 2 (FGF2); transforming growth factor β (TGFβ) and other growth factors and cytokines, such as ILs, interleukines [15,16,40,41,42,43,44,45,46,47,48,49,50,51,52,53].

Regarding the pituitary, there are several proposed mechanisms explaining estrogen action on the development of lactotropic hyperplasia, hyperprolactinemia and prolactinoma (summarized in Figure 1). They include direct and indirect effects of estradiol on already-differentiated lactotropic cells to enhance their proliferative and secretory activity and potentiated transdifferentiation from another lineage-related hormone-producing cell type, as well as stimulation of pituitary stem/progenitor cell differentiation in the direction of a lactotropic cell line. Moreover, estrogen may induce changes in the cellular microenvironment by contributing to the recruitment of immune cells and affecting the synthesis of pituitary growth factors and cytokines, thus contributing to tumor initiation and promotion. Effects of estrogen on the hypothalamic regulation of PRL cells (including TRH, dopamine, somatostatin, and other signaling pathways) have been, inter alia, described by other authors [39].

## 3. Effects of Estradiol on Differentiated Lactotropic Cells in the Anterior Pituitary

Pituitary lactotropic cells of both sexes are typical estrogen-responsive cells. Estrogen treatment in vivo and in vitro induces their proliferation and PRL secretion (Figure 1), while long-term treatments lead to the formation of PRL-secreting tumors in sensitive rodent strains [3,40,54]. The rapid development of prolactinoma in response to prolonged estrogen treatment makes rodent models of sensitive strain useful for studying tumorigenesis. 

**Figure 2:**
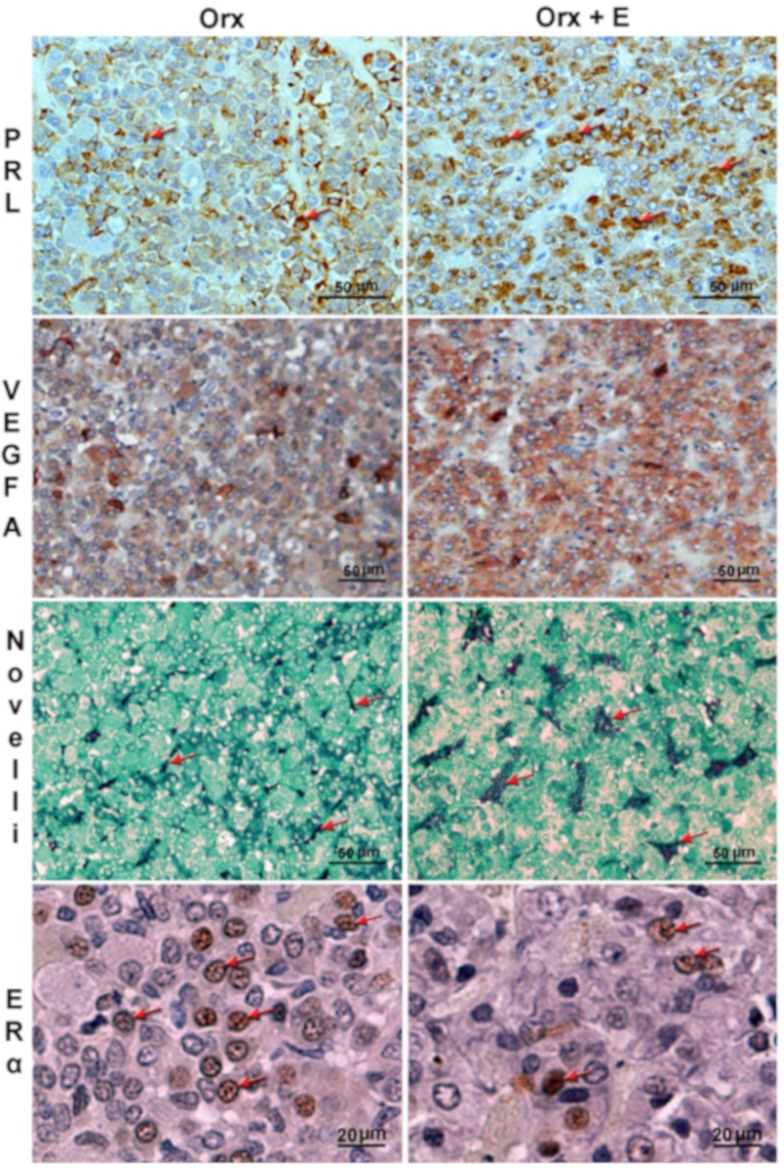
Representative micrographs of immunohistochemically (IHC) or histochemically stained anterior pituitary sections of orchidectomized (Orx) and estradiol-treated orchidectomized (Orx+E) middle-aged rats. Hypertrophy and hyperplasia of prolactin-immunopositive cells (PRL, red arrows; adopted from our previous publication [55] and reprinted by permission of the Licensors - publishers Elsevier (Licence number 4774000297301)), stronger vascular endothelial growth factor A (VEGFA) immunopositivity, more abundant blood vessels (red arrows, Novelli histochemical staining) and lower nuclear estrogen receptor α immunopositivity (red arrows, ERα), respectively, are observable after estradiol treatment. Micrographs were obtained according to the same procedures described in our earlier papers [55]. Briefly, for IHC characterization of anterior pituitary tissue, the primary rabbit antisera directed against PRL (Abcam, Cambridge, UK; 1:200), VEGFA (Abcam, Cambridge, UK; 1:100) or ERα (1:100; Santa Cruz Biotechnology), were applied overnight at 4 °C. Swine anti-rabbit IgG- horseradish peroxidase (HRP; Dakopatts, Glostrup, Denmark; 1:100) was applied as a secondary antiserum for 1 h, while visualization was performed using diaminobenzidine tetrahydrochloride (DAB; Dakopatts, Glostrup, Denmark) at concentrations suggested by the manufacturer. Sections were counter-stained with hematoxylin and mounted in DPX medium (Sigma-Aldrich, Barcelona, Spain). For Novelli histochemical staining, sections were incubated in hot 1 N HCl (60 °C, 3 min), followed by staining in 1% acid fuchsin (Fluka Chemie AG, Buchs, Switzerland; 30 s) and 1% light green (Sigma-Aldrich, St. Louis, MO, USA; 3 min), respectively. In between, the slides were washed in PBS (for Nowelly, distilled water), and after the last step, mounted in DPX (Sigma-Aldrich, Barcelona, Spain). Scale bar is shown in the right corner.

We examined effects of male estrogenisation on the pituitary and downstream elements of the endocrine system using orchidectomized (Orx) Wistar rats of young-adult- (2–3 months old) and middle age (15–16 months old) [55,56]. The administered dose of estradiol dipropionate (s.c. injection of 0.0625 mg/kg b.w./day during three weeks; Orx+E) had a beneficial bone-protective effect [57]. Irrespectively of age, estradiol treatment induced a significant increase in pituitary weight [55,56], indicating preserved estrogen sensitivity with advancing age. Hypertrophy and hyperplasia of PRL-immunopositive cells were observed in Orx+E middle-aged males [55], as illustrated in Figure 2. Increased immunohistochemical (IHC) expression of vascular endothelial growth factor A (VEGFA), a promoter of angiogenesis, and more prominent vasculature were clearly visible upon estradiol treatment (Figure 2, Table 1). 

In human and rodent pituitary, nuclear estrogen receptor (ER) α plays a major role in the physiological regulation of lactotropic-cell secretory and mitotic activity, whereas in growth hormone (GH)-producing cells, both ERα and β are of importance [58,59]. With regard to sex-related differences, the number of lactotropic cells and the expression and secretion of PRL are higher in females than in males, both in rodents and humans [41,60,61]. Analyses of a transgenic mouse line that lacks androgen receptors in the pituitary gland showed that androgens and androgen receptor (AR)-mediated signaling in the pituitary also contribute to PRL maintenance and suppression of abnormal PRL production in males [62]. Fletcher et al. [16] used transcriptomic (and other relevant) analyses in rats and showed that lactotropic cells contain the largest number of sex-dominant genes. Interestingly, these authors reported mRNA and protein expression of retinoic-acid-synthesizing enzyme aldehyde dehydrogenase 1A1 (ALDH1A1 or RALDH1), only in lactotropic cells from males but not from females, and in folliculo-stellate cells (FS) from both sexes [16]. This enzyme catalyzes the conversion of 9-*cis*-retinal to 9-*cis*-retinoic acid, which increases the expression of dopamine receptor type 2 (DR2) in normal and pituitary tumors [63,64]. In line with this, promotion of lactotropic cell secretion and proliferation in adults is associated with reduced expression of retinoic-acid-synthesizing enzyme ALDH1, indicating its involvement in this process. Interestingly, ALDH1 was reported to have AR binding properties [65], but at the same time, its gene and protein expression were reported to be downregulated by 17β-estradiol treatment (Figure 1) via ERα pathway in male rodents [42]. Further research of estrogen–androgen interconnections may explain some of the complexities of regulation of lactotropic cells observed in the male.

**Figure 3:**
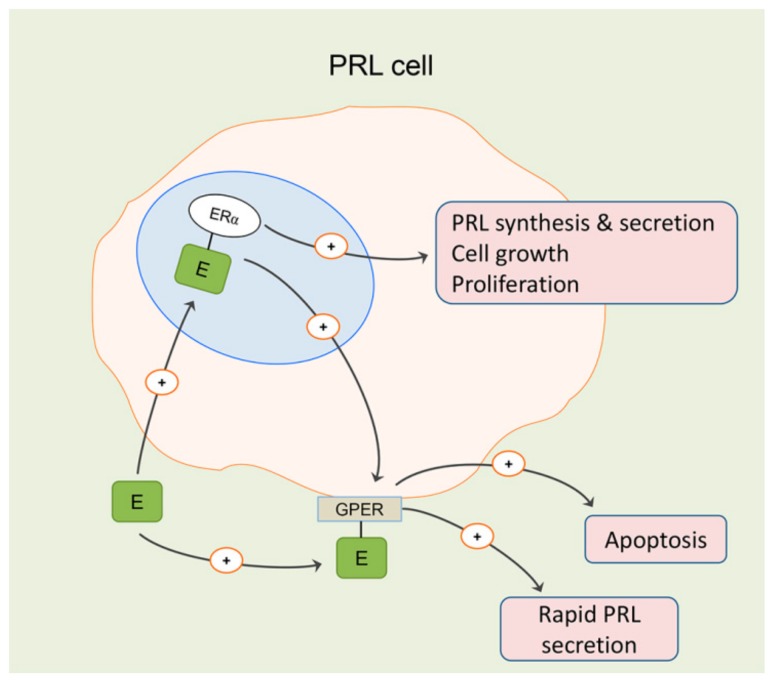
Physiological and pathophysiological mechanisms of estrogen (E) action on proliferation, growth and secretion of lactotropic (PRL) cells are probably largely mediated by nuclear estrogen receptor (ER) α. Membrane G-protein-coupled estrogen receptor 1 (GPER)-mediated estrogen signaling seems to be more involved in antiproliferative and apoptotic actions in the pituitary and may contribute to rapid secretion of PRL under physiological conditions. Its expression is under ERα-mediated nuclear signaling [15,59,68,69,70].

Aside from its normal physiology, ERα-mediated signaling is important for development of the pituitary prolactinomas and may also be involved in increased aggressiveness of lactotropic tumors in men [15] (Figure 3). Estrogen was the first-discovered inducer of pituitary tumor transforming gene (*Pttg*) through its nuclear ERα, a well-known proto-oncogene whose aberrant accumulation is known to cause genetic instability that could underlie cancer development [43] (Figure 1)**.** PTTG interacts with a wide variety of genes and molecules related to survival, mitogenesis, tumor growth and invasion [66]. It is also expressed in the majority of human pituitary adenomas, and its expression parallels that of Ki-67, while both are correlated to a more aggressive behavior of pituitary adenomas [67]. PTTG stimulates fibroblast growth factor 2 (FGF2) and VEGF production, thus further promoting the invasiveness and angiogenesis of pituitary adenomas, especially prolactinoma and growth-hormone-secreting adenomas [43,44] (Figure 1). 

Pituitary adenomas are generally less vascularized than normal pituitary tissue, though a significantly higher degree of vasculature, also observed in our estrogenized Orx males (Figure 2), has been shown in macroprolactinomas when compared with noninvasive microprolactinomas [71]. Male patients typically have higher serum PRL levels and larger tumor volumes, exerting more aggressive and/or refractive behavior in comparison with female patients [10,72,73]. Some authors indicate higher [74], while others lower [11], expression of ERα in prolactinomas from male patients. In our Orx middle-aged model, a lower nuclear ERα IHC signal was also observed in the anterior pituitaries upon estradiol treatment (Figure 2). 

Wierinckx and associates [15] used the most advanced methods (including transcriptomic, microarray and comparative genomic hybridization analyses) to examine potential sex-linked dysregulation in aggressive lactotropic tumors that are less sensitive to dopamine agonists in men. Aside from low expression of ERα, some genes from the X chromosome (*Ctag2*, *Fgf13* and *VEGFD*) and chromosome 19p (*STAP2*) were found to be upregulated in lactotropic tumors, only in men [15]. Cancer testis antigen (*Ctag2*), whose expression correlated with various markers of aggressiveness in male prolactinomas, is implicated in the aggressive behavior of breast cancer [75] and repression of estrogen signaling [76]. Moreover, the authors [15] hypothesized that upregulated expression of FGF13 enhanced survival of cancer cells [77] and underlined that not only *Vegfd*, but a significant number of proteins involved in promotion of angiogenesis, were upregulated exclusively in male prolactinomas. Finally, they indicated that chromosome abnormalities, in particular 19p gain, may underly more aggressive behavior of prolactinoma in men and pointed to the mechanism through which lower expression of ERα in male prolactinomas may increase the transcript level of *STAP2* and consequently, through interaction with the STAT signaling pathways, contribute to genome instability and tumor progression [15]. 

Mitogen-activated protein kinase (MAPK) signaling abnormalities are strongly related with pituitary adenoma development, and changes in molecules such as ERK, p38, JNK, Ras, Akt, NF-kB and TNF are identified as the most important [78]. In addition to nuclear receptors, membrane-bound and G-protein-coupled estrogen receptor 1(GPER), which subsequently activates pathways such as MAPK, plays an important role in rapid estrogen actions. However, the role of membrane-bound ERs seems to be rather complex, as antiproliferative [68] and apoptotic [69] actions of estradiol mediated by these receptors have been reported in the pituitary (Figure 3). Thus, even femtomolar doses of endogenous 17β- and synthetic 1α-ethinil-estradiol activated MAPK, ERK and JNK pathways in membrane estrogen receptor-enriched GH3/B6 pituitary tumor cells, though the activation of the p38 MAPK pathway required nanomolar doses of estrogens [69]. Fulvestrant, a pure ER antagonist that blocks the nuclear ERs, as well as cytoplasmic and membrane-bound ERs [79], significantly suppressed cell viability and invasion of rat GH3 cells by simultaneous regulation of ERK1/2, JNK1/2 and p38 MAPK signaling pathways [45] (Figure 1). GPER is mainly responsible for the rapid nongenomic effects of estrogen, which are associated with activation of ERK and AKT, as well as for a rapid increase in intracellular calcium levels [80,81]. GPER is expressed ubiquitously and has diverse biological effects, including vascular hypertrophy, regulation of cell growth, migration and apoptotic cell death [82]. However, the estradiol-mediated effect on proliferation of lactotropic cells in primary culture seems not to be due to GPER signaling [83]. The physiological roles of GPER in the pituitary are still not completely understood, but it seems to be involved in modulation of secretion of gonadotropins [84] and PRL [70] (Figure 3). At the same time, expression of the GPER gene is under estrogen-mediated genomic signaling [70] (Figure 3). 

## 4. Effects of Estradiol on the Cellular Microenvironment in the Anterior Pituitary

Limited data exist on estrogen-induced changes of the cellular microenvironment in the pituitary, as well as on the tumor microenvironment of pituitary adenomas. The tumor microenvironment influences tumor behavior and aggressiveness and includes immune cells, fibroblasts, endothelial cells, extracellular matrix and numerous secreted factors such as cytokines and growth factors. 

FS cells also express ERs [85]. This is a heterogeneous cell lineage that serves various functions, including structural, signaling and a supportive role to hormone-producing cells [86]. Besides their importance for normal anterior pituitary physiology, their identification in pituitary tumors and the tumor microenvironment suggests that FS cells may also have some major implications in these tumors, but the exact roles remain to be elucidated [87]. 

S100β is generally accepted as a marker gene for FS cells [88]. Comparison of the total number of S100β-immunopositive cells in the pituitaries of estrogen-sensitive Fischer-344 with the same parameter in the insensitive Sprague-Dawley rat strain revealed that ovariectomized F344 rats have significantly more S100β-immunopositive cells than ovariectomized Sprague-Dawley females [89]. The interstrain variation in PRL cell responsiveness to estrogens has also been examined in genetic studies. Genetic variants that reside within *Ept7* (estrogen-induced pituitary tumor, a quantitative trait locus mapped to rat chromosome 7 that is orthologous to an interval within the 8q24.21 region of the human genome, associated with risk of numerous cancer types and other common diseases) have been implicated in lactotropic cell responsiveness to estrogens [90]. In particular, *Ept7* locus carries Myc, a well-known proto-oncogene, and estrogens enhance *Myc* expression in the rat anterior pituitary gland [46] (Figure 1).

In addition, FS cells produce numerous cytokines and growth factors, including interleukin-6 (IL-6), follistatin, basic fibroblast growth factor, transforming growth factor β (TGF β), VEGF and leukemia inhibitory factor, which all regulate lactotropic cell proliferation and prolactinoma development [47]. The TGFβ1 expression was reduced in human and animal prolactinomas [91,92]. Estradiol inhibits most of the components of the TGFβ1 system, and this mechanism seems to be of importance with regard to the balance and negative control of the function of lactotropic cells [40] (Figure 1). Sex-related differences in the pituitary TGFβ1 system were identified in different models of prolactinoma, with the authors proposing that the higher levels of the TGFβ1 system in males may contribute to a lower proliferation rate in males compared with females, under physiological conditions [93]. The functional crosstalk between immune signals and the ER at the protein level may be important for both physiological states and prolactinoma development. Such inter-relation was reported for the ER, morphogenetic protein-4 (BMF-4), a member of the TGF-β superfamily, and Smad-4 in pituitary prolactinoma [94].

Aside from the effect of estrogen on FS cells, Fujiwara et al. [95] identified a remarkably increased number of alternatively activated (M2) macrophages upon diethylstilbestrol application in comparison with normal pituitaries (Figure 1). Interestingly, recruitment of M2 macrophages was detectable even before tumor formation, indicating their role in tumorigenesis [95]. Recent study results also indicated that an increased number of M2 macrophages correlated with microvessel density, indicating their role in angiogenesis and vasculature modulation in pituitary adenomas [48]. Furthermore, tumor-associated fibroblasts produce cytokines and may increase tumor aggressiveness and proliferation [96], but the effect of estrogen on this cell population in the pituitary is still largely unknown.

## 5. Effects of Estradiol on Stem/Progenitor Cells and Transdifferentiation of Lineage-Related Hormone-Producing Cells in the Anterior Pituitary

In addition to the effect on proliferative and secretory capacity of already-differentiated lactotropic cells, estradiol might induce their differentiation from the postnatal pituitary stem/progenitor (SP) cells [49] (Figure 1). The SP cells are present in both rodent and human pituitary until late adult life and have preserved their capability to self-renew and differentiate, giving a contribution to all anterior pituitary lineages [97]. 

**Figure 4:**
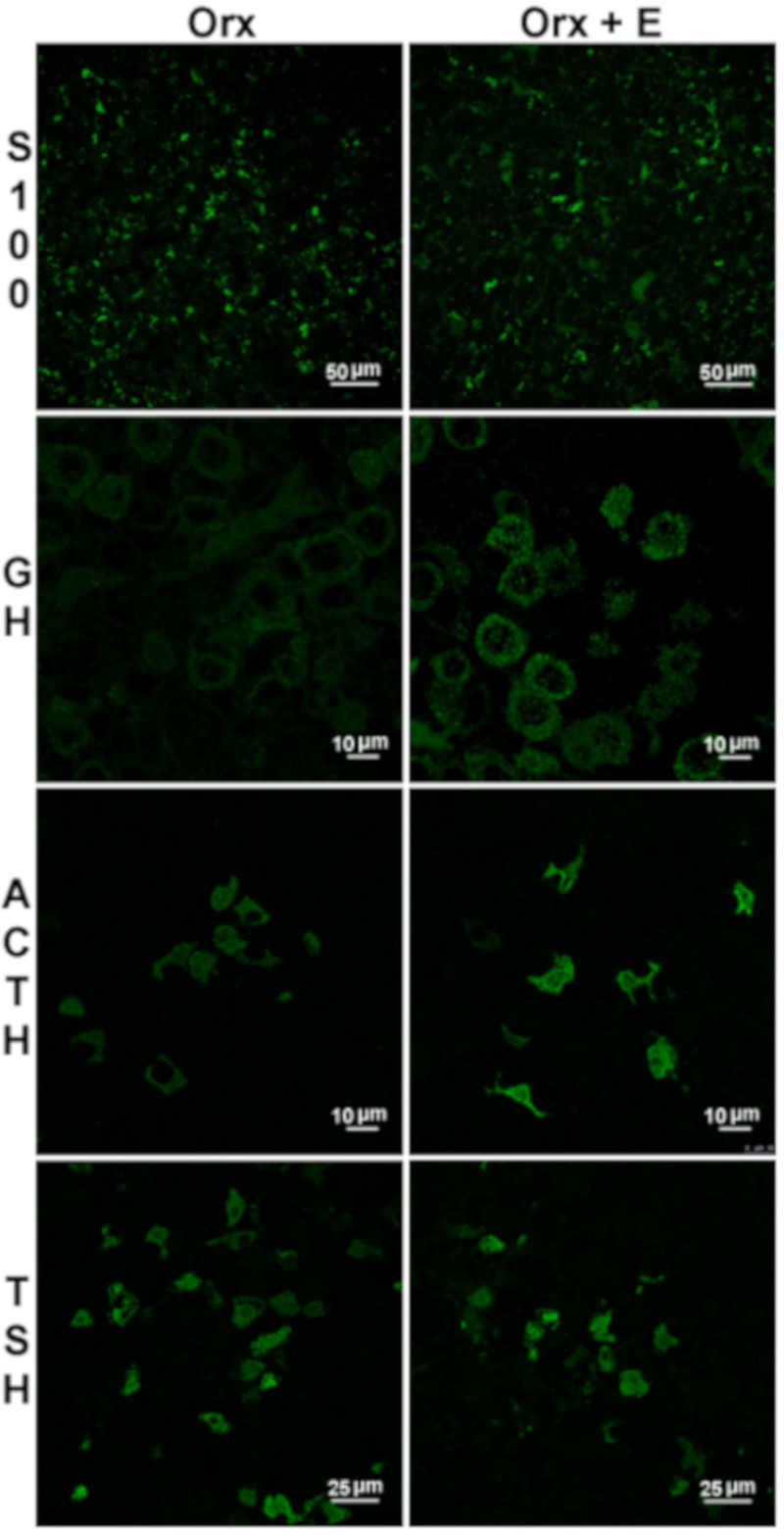
Representative micrographs of immunofluorescently (IFC) stained anterior pituitary sections of orchidectomized (Orx) and estradiol-treated orchidectomized (Orx+E) middle-aged rats. Weaker immunofluorescent signal of S100β, stronger growth hormone (GH; adopted from our previous publication [99] and reprinted by permission of the Licensors - publishers Springer (Licence number 4774320912101)) and adrenocorticotropic hormone (ACTH) immunofluorescent signal, and unchanged immunofluorescent signal of subunit β of thyroid-stimulating hormone (TSHβ; adopted from our previous publication [55] and reprinted by permission of the Licensors - publishers Elsevier (Licence number 4774000297301)), respectively, are observable after estradiol treatment. All figures were obtained according to the same procedure described in our earlier papers [55,99]. Briefly, for IFC staining, the following primary antibodies were applied overnight at 4 °C: mouse monoclonal anti-S100β antibody (Abcam, Cambridge, UK; 1:100), polyclonal rabbit anti–rat TSHβ, GH and ACTH (donation from Dr. A. F. Parlow, National Institute of Health, Bethesda, MD, USA; dilutions 1:500, 1:200, 1:200, respectively). Alexa Fluor 488 donkey anti-rabbit IgG (Invitrogen Life technologies, CA, USA; 1:300) was applied as secondary antiserum for 1 h. The sections were mounted with Mowiol 4–88 (Sigma-Aldrich, St. Louis, MO, USA). Scale bar is shown in the right corner.

SOX2 is generally accepted as the primary marker for SP cells and is often coexpressed with SOX9, with partial overlap of S-100β expression [50,51] (Figure 1). Estrogen-induced changes in S100β-immunofluorescent signal indicates a decreased signal in comparison to Orx middle-aged males (Figure 4). Our data, together with finding that FS cells coexpress S100β and SOX2, are in line with the assumption that a subpopulation of FS cells also serves a pool of SP cells [16,51,98]. SOX2^+^/ SOX9^+^ cells were reported to proliferate due to the effect of estradiol in vitro, with a predisposition to become lactotropic and luteinizing cells, with a small number differentiating into GH cells [49] (Figure 1). However, the number of differentiated cells produced is small, indicating low levels of physiological turnover, therefore indicating that proliferation of differentiated cells is the most likely source of pituitary phenotypic plasticity and adaptability to large variations [97]. 

Su et al. [37] analyzed the expression pattern of tumor stem-like cells isolated from MMQ rat prolactinoma cells and found that *Bcl 2, VegfA, Pten, Jun, Fos, and Apc2* gene expressions were upregulated and the expression of Myc was downregulated in these cells. The model of cancer stem cell proposes that a small population of cells with stem-like properties initiates and maintains tumorigenesis and may be more resistant to therapy than other tumor cells [100,101]. The resistance is probably due to their often slow cycling, nonproliferative states [102] and/or the expression of ABC transporters, and high levels of aldehyde dehydrogenases [100].

The occurrence of bi- and multi-hormonal cells in the normal anterior pituitary has been described both in rodent models and in humans [103,104], indicating a more complex structural organization associated with cellular expansion, transdifferentiation (functional phenotypic conversion without cell division), and pituitary homeostasis, physiological plasticity and tumorigenesis [41].

Pituitary specific-lineage transcription factors are involved in the cell-specific expression and regulation of the gene products of hormone-producing cells. These transcription factors can also be used to characterize pituitary tumors such as Pit-1 (Pit-1 lineage including thyrotropic, somatotropic, and lactotropic cells) or ERα (lactotropic and gonadotropic lineage) [13]. According to the currently accepted model, Pit-1 positive cells during development differentiated into TSHβ-producing and GH/PRL dual-expressing somatolactotropic cells [105]. The somatolactotrope precursor then gives rise to the terminally differentiated somatotropes and lactotrops, which produce high levels of GH and PRL protein, respectively [106]. Moreover, it was reported that a small population of bihormonal PRL/GH cells (lacto-somatotrophs) remains present in the pituitaries of adult mice [41,52,53] (Figure 1). Despite significant transcriptomic similarity, almost 300 genes have been identified to be crucial to the ability of GH and lactotropic cells to respond to the appropriate physiological signals to secrete their respective hormones [14]. 

Bihormonal PRL/ GH cells were more abundant in the pituitary of females than in males, indicating that transdifferentiation contributes to adaptation of the pituitary toward hormonal fluctuations during the menstrual (estrous) cycle, pregnancy and lactation [53]. In addition to this, recent single-cell transcriptomic and other relevant analyses of adult mouse pituitaries demonstrated a multihormonal cluster of hormone-producing cells that, aside from Pit-1 lineage (PRL, GH and TSHβ), co-expressed LHβ and proopiomelanocortin, a precursor of ACTH [41] (Figure 1). Cells from this cluster have great phenotypic plasticity potential, which enables rapid response to physiological demands [41]. 

Though we have not examined PRL/GH or other bi- and multihormonal modalities in the pituitary, it is possible that processes of terminal differentiation from SP cells and/or transdifferentiation from lineage-related cells contributed to lactotropic cell hyperplasia observed in the estrogenized Orx male rats. Hovewer, the total number of GH-immunopositive cells was not significantly changed upon estradiol treatment. On the other hand, GH immunofluorescence in the pituitary and serum GH concentrations were elevated [99], in line with combined PRL and GH secretion phenotype of prolactinoma (Figure 4 and Table 1). 

## 6. Effects of Estradiol on Pituitary ACTH Cells and Glucocorticoid Homeostasis in Males: Possible Implication on Prolactinoma Development 

It is known that sex differences exist in some aspects of hypothalamus—pituitary-adrenal (HPA) axis activity. Women are more susceptible to autoimmune disease development and stress-related psychiatric disorders, while men have a greater risk of infectious diseases and nonreproductive cancers [107,108], pathologies that more or less include HPA axis involvement. However, organization of the stress-responsive HPA axis is highly conserved throughout mammalian phylogeny [109], and there is very little sexual dimorphism in the pattern of ACTH cell gene expression [16]. 

In estradiol-treated Orx middle-aged rats cytomorphology of ACTH cells was generally preserved and characterized by elongated or round, sparse ACTH cells that were leaning on the capillaries [110]. Moreover, in addition to increased PRL and GH, concentrations of intrapituitary and serum ACTH were markedly increased, while circulating corticosterone levels remain unchanged [110], as presented in Figure 4 and Table 1. 

A physiologically relevant dose of estradiol (10 nM) was found to stimulate CRH gene expression as well as to increase IL-6 mRNA and protein levels [111], which clarifies the agenda of molecular events underlying the rise of estradiol-caused CRH gene expression in the hypothalamic paraventricular nucleus (PVN). Considering the role of hypothalamic IL-6 in stimulation of CRH gene expression [112], it is possible that IL-6 elevation upon estradiol treatment significantly contributes to the CRH secretion. 

When it comes to clinical estradiol application in men, it was reported that polyestradiol phosphate (Estradurin; 80 mg i.m., once a month, during 12 months) did not change circulating ACTH concentrations in aged prostate cancer patients, neither alone nor in combination with orchidectomy [113]. In patients with disseminated prostate cancer treated with Estracyt (estradiol normustine phosphate), either subnormal or low-normal plasma ACTH concentrations followed by high cortisol levels were observed [114]. Mental-stress-induced increases in blood ACTH, cortisol and adrenaline levels were attenuated after estradiol supplementation in aged hypogonadal men [115]. It has been recently reported that transsexual male patients subjected to estradiol treatment have increased ACTH and cortisol output in response to CRH [116]. Moreover, the application of estradiol valerate in transsexual male patients led to initial, moderate decreases of ACTH and cortisol blood levels, and subsequent significant increases of both parameters, coincident with the time when circulating estradiol concentrations become nondetectable [117].

**Figure 5:**
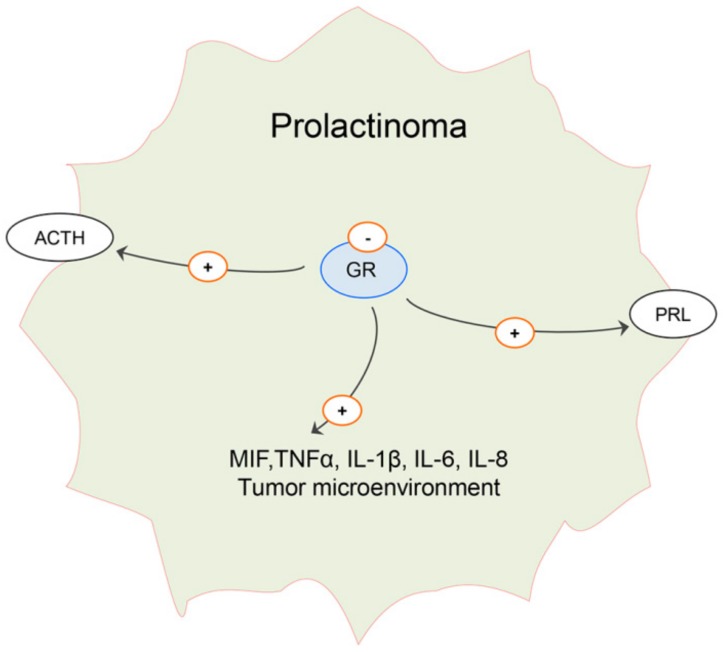
Hypothetic role of attenuated glucocorticoid hormone signaling in prolactinoma. Expression of glucocorticoid receptor (GR) is decreased in estrogen-induced prolactinoma and may contribute to increased adrenocorticotropic hormone (ACTH) and prolactin (PRL) secretion, as well as to changes in local secretion of growth factors and interleukins such as mitogen inhibiting factor (MIF), tumor necrosis factor α (TNFα), interleukins (IL) 1β, 6 and 8, thus promoting “tumor-friendly“ microenvironment [110,118,119,120].

Glucocorticoids normally suppress PRL secretion, while it was established that estrogen-induced pituitary tumors in rats, predominantly composed of proliferating lactotropic cells, express low content of glucocorticoid receptors (GR) [118]. Binding of radiolabeled, synthetic glucocorticoid dexamethasone to soluble GR is several times lower in estrogen-induced pituitary tumors than in normal pituitaries [118]. Consequently, glucocorticoid negative feedback on synthesis, and consequently, secretion of PRL is deficient in pituitary-tumor-bearing rats [118]. Regardless of the blood levels of glucocorticoids (variable in different studies with estradiol, as stated previously), reduced expression of GR in estrogen-induced pituitary tumors suggests generally weak glucocorticoid input in this context. Even ACTH-secreting pituitary tumors express a largely autonomous mode of ACTH secretion that is supposedly insensitive to physiological negative feedback and glucocorticoid levels [119]. On the other hand, glucocorticoids stimulate secretion of macrophage migration inhibitory factor (MIF) by folliculo-stellate cells, macrophages and T lymphocytes. MIF has been identified in the pituitary gland, and this counter-regulates the inhibitory effects of glucocorticoids on TNFα, IL-1β, IL-6 and IL-8 production [120]. Therefore, attenuated glucocorticoid signaling may actually promote prolactinoma development and/or progression by affecting its cellular microenvironment. Possible changes in glucocorticoid signaling that may contribute to prolactinoma progression are illustrated in Figure 5.

## 7. Effects of Estradiol on TSH Cells in Males and Putative Changes in Local Thyroid Hormone Metabolism and Action in Prolactinomas

Sex steroids affect the feedback response of TSH cells to free thyroid hormone concentrations in blood diversely in male and female rodents [121,122]. Male rats have higher TSH values and are more prone to develop thyroid diseases (or even thyroid carcinoma) in response to goitrogenic agents compared with females [121,122]. In humans, sex-related differences in TSH values are not as clearly visible as in rodents, but women are more prone to thyroid dysfunctions in comparison with men. Moreover, clinical results indicate a negative relationship between the free l-triiodothyronine (T_3_) resistance index and age in men, while there were no apparent relationships in women [123]. Species-specific features of the sex-related regulation of pituitary TSH may be explained by differences in the functional anatomy of thyroid hormone feedback, with a particular emphasis on differential expression of deiodinase enzyme (Dio). Namely, deiodinase type 1 (Dio 1) is expressed in rat, while deiodinase type 3 (Dio 3) is expressed in human anterior pituitary. Deiodinase type 2 (Dio 2) is expressed in TSHβ cells in rats [124], while in humans it seems to be expressed only in FS cells [125,126].

When we tested how pituitary-thyroid-periphery signaling responds to orchidectomy in our middle-aged rat model, unchanged serum l-thyroxine (T_4_) and TSH were detected [127]. On the other hand, liver Dio 1 and pituitary Dio 2 enzyme activities were decreased, which indicated a local hypothyroid state, in line with altered cytomorphology of thyrotropic cells, which appeared enlarged and vacuolated, as in hypothyroid states [122]. In addition to this, most recently, specific cell-type transcriptomic analysis showed that 100% of sex-dominant genes in TSH cells were male-dominant [16]. Our further research confirmed that neither significant change in TSHβ immunofluorescence in the pituitary, nor serum concentration of TSH, were detected upon estrogenisation of Orx rats [55], as presented in Figure 4 and Table 1. Similar results were obtained in male-to-female transsexual patients exposed to long term treatment with antiandrogen plus estrogen [128]. However, pituitary hyperplasia and increased TSH production together with PRL cell recruitment may also be induced by primary hypothyroidism, as was noted in some clinical cases [129,130]. 

**Figure 6:**
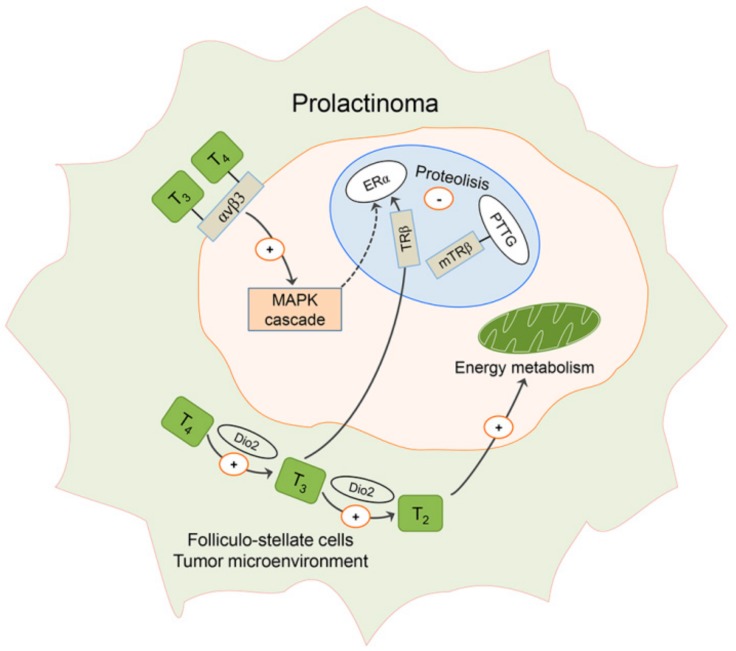
Hypothetic role of enhanced thyroid hormone signaling in prolactinoma. Deiodinase type 2 (Dio 2) expression in folliculo-stellate cells is increased in prolactinomas, which may lead to increased production of l-triiodothyronine (T_3_) and 3,5-T_2_ (T_2_) from l-thyroxine (T_4_) in the pituitary_._ T_3_- thyroid receptor (TR) β complex prevents proteolisis of estrogen receptor (ER) α in the cell nucleus, while mutated thyroid receptor (mTRβ) binds to pituitary tumor transforming gene (PTTG), thus preventing its proteolysis. Both T_4_ and T_3_ may bind to its membrane integrin αvβ3 receptor and activate mitogen-activated protein kinase (MAPK) cascade and phosphorylation processes, including ERα phosphorylation (dashed arrow). Increased concentration of T_2_, demonstrated in pituitary adenomas, may contribute to progression of prolactinoma through stimulation of energy metabolism in mitochondria [132,133,134,135,136,137,138,139,140].

It is largely unknown whether genetic and/or environmental alterations in (local) thyroid hormone metabolism and action may contribute to pituitary adenoma development and its aggressiveness. However, a large body of evidence suggests that subclinical and clinical hyperthyroidism both increase the risk of breast and lung cancers [131], and an association exists between prolactinoma development and family history of these diseases [8]. Deregulation of thyroid hormone metabolism and action may result in major disturbances of cellular physiology such as those observed in tumorigenesis. Expression of Dio 2 seems to be variable in pituitary tumors: Dio 2 mRNA levels were increased in different types of pituitary adenoma, including TSH- and PRL-producing adenomas, with unchanged or lower Dio 1/Dio 2 ratios found among patients with similar types of tumors [132,133]. Thus, it seems probable to assume that increased expression of Dio2 may lead to increased intrapituitary production of T_3._ Hypothetic alterations in thyroid hormone metabolism and action in prolactinoma are presented in Figure 6. 

In this context, it may be important to know that T_3_- TRβ receptor complex prevents proteolysis of estrogen receptor-α (ERα) in lactotropic cells [134], while mutated TRβ receptor, which lost the ability to bind T_3_, prevents degradation of PTTG in thyroid cancer [135] (Figure 6). Moreover, nongenomic thyroid signaling mediated through the membrane integrin αvβ3 receptor [136,137] promotes tumor cell proliferation and angiogenesis via stimulation of MAPK and phosphorylation processes, including phosphorylation of ERα [138] (Figure 6). Upregulated expression of αvβ3 occurred in the parenchyma of a subset of human pituitary adenomas but not in normal pituitaries [136]. 

To the best of our knowledge, there are still no data regarding the putative association between changes in thyroid hormone metabolites and the pituitary adenoma or prolactinoma, especially upon estradiol treatment. As a part of TH metabolism, Dio enzymes are proposed to catalyze the production of physiologically active 3,5-diiodothyronine (3,5-T_2_) [139]. 3,5-T_2_ is present in significant concentrations in human serum [141] and seems to have important implications in the regulation of energy metabolism, with a direct effect on mitochondria as the most likely mechanism [139,141,142,143] (Figure 6). So far, serum 3,5-T_2_ concentrations have been associated with some pathophysiological states in humans, mainly related to glucose and lipid metabolism [144]. This metabolite exerts thyromimetic activity, suppresses TSH and stimulates GH [145,146,147], though not as strongly as T_3_ [139]. Moreover, the local concentration of 3,5-T_2_ in human brain tumors and pituitary adenomas is significantly increased in comparison with nontumoral regions of the human brain [140]. Further experimental research and analyses are needed to shed more light on this still largely unexplored area.

## 8. Conclusions

We should be aware that estradiol exerts a rather complex regulatory pattern in the anterior pituitary, affecting hormone secretion, cell proliferation and death at the same time, as well as the cellular microenvironment. A significant number of genes implicated in genesis and progression of prolactinoma, such as *Pttg*, *Myc*, MAP kinases, *Vegf* and other growth factors and cytokines, are regulated by estrogen signaling in both sexes. Development of macroprolactinomas with more aggressive features in men in comparison to women may be associated with attenuation of ERα-mediated control and accumulated chromosomal abnormalities. Symptomatic estrogenization of men bears a certain risk of tumor development in genetically susceptible patients, with probable effect on other hormone-producing cells and local hormone signaling. Studies with rodent models should provide the identification of new molecular targets and additional treatment modalities for patients with prolactinomas resistant to the classical therapeutic approach. Further examinations of alterations in local glucocorticoid and thyroid hormone metabolism and actions upon estradiol treatment may shed more light on tumor microenvironment and energetic pathways in the pituitary adenoma. 

## Figures and Tables

**Table 1:** The effects of estradiol application on relevant anterior pituitary parameters in orchidectomized middle-aged rats (Orx).

Measured Parameter	Orx+E vs.Orx
Pituitary weight	↑
VEGFA immunopositivity	↑
PRL immunopositivity	↑
ERα immunopositivity	↑
S100β immunopositivity	↓
Pituitary GH immunopositivity	↑
Pituitary ACTH immunopositivity	↑
Pituitary TSH immunopositivity	↓
Serum PRL	↑
Serum GH	↑
Serum ACTH	↑
Serum TSH	n.s.
Serum corticosterone	n.s.
Serum L-thyroxine	n.s.

VEGFA, vascular endothelial growth factor A; PRL, prolactin; ERα, estrogen receptor α; GH, growth hormone; ACTH, adrenocorticotropic hormone; TSH, thyroid-stimulating hormone.
